# Quantifying synergistic interactions: a meta-analysis of joint effects of chemical and parasitic stressors

**DOI:** 10.1038/s41598-023-40847-6

**Published:** 2023-08-22

**Authors:** Nina Cedergreen, Kathrine Eggers Pedersen, Brian Lund Fredensborg

**Affiliations:** Department of Plant and Environmental Sciences, University of Copenhagen, Thorvaldsensvej 40, 1871 Frederiksberg, Denmark

**Keywords:** Ecology, Environmental sciences

## Abstract

The global biodiversity crisis emphasizes our need to understand how different stressors (climatic, chemical, parasitic, etc.) interact and affect biological communities. We provide a comprehensive meta-analysis investigating joint effects of chemical and parasitic stressors for 1064 chemical-parasitic combinations using the Multiplicative model on mortality of arthropods. We tested both features of the experimental setup (control mortality, stressor effect level) and the chemical mode of action, host and parasite phylogeny, and parasite-host interaction traits as explanatory factors for deviations from the reference model. Synergistic interactions, defined as higher mortality than predicted, were significantly more frequent than no interactions or antagony. Experimental setup significantly affected the results, with studies reporting high (> 10%) control mortality or using low stressor effects (< 20%) being more synergistic. Chemical mode of action played a significant role for synergy, but there was no effects of host and parasite phylogeny, or parasite-host interaction traits. The finding that experimental design played a greater role in finding synergy than biological factors, emphasize the need to standardize the design of mixed stressor studies across scientific disciplines. In addition, combinations testing more biological traits e.g. avoidance, coping, and repair processes are needed to test biology-based hypotheses for synergistic interactions.

## Introduction

### Background

The world biodiversity crisis has emphasized our lack of understanding of how different stressors and their interactions affect species populations and communities. During the last decade the importance of combined exposure to chemical and parasitic stressors, including pathogenic viruses, fungi and bacteria, for organism health has gained increasing recognition^1–4^. Attention has mainly been devoted to improve methods for controlling pests in agricultural settings^5^, for controlling insect vectors of human diseases^6^, and for reducing mortality in beneficial insects^7–10^. Hence, it is well known, that one stressor may render the organism more sensitive to a second stressor and that environmental pollutants may interact with natural infections resulting in effects not readily predictable from neither the ecology of the organism and its parasite, nor the inherent toxicity of the pollutant^11^. The lack of predictability, however, is largely due to the lack of a consistent use of validated conceptual models to predict organism response to combinations of chemicals and parasitic stressors. Using a model describing the expected effects of a combination based on knowledge of the effect of the single stressors when applied individually, makes it possible to define if effects are larger than predicted, often termed synergy, or smaller than predicted, termed antagony, or simply follow the model and therefore are predictable.

Previous meta-analyses have investigated the frequency of synergistic interactions between mixtures of chemicals using the reference model of Concentration Addition^12–15^, which is the regulatory default model within the areas of (eco)toxicology and chemical stress^16^. There is, however, a lack of consensus of which model to use when it comes to multiple stressors beyond the field of chemical stressors^17–22^. An increasing number of studies of combined exposure to (agro)chemicals and parasites claim synergistic effects, but the reference model used to define synergy (and whether a reference model is used at all) varies^1,9,23^. Hence, no meta-analyses of combined chemical and parasitic stressor across scientific disciplines ranging agronomy to ecology using a consistent model framework exist.

Understanding how models can be used to predict the effect of multiple stressors, is essential in ecology to predict joint effects of multiple stressors, and to quantify the proportional effect of individual stressors in a multi-stressor scenario, thereby identifying key stressors or stressor interactions threatening local biodiversity^17,20,24,25^. In addition, quantifying and ideally understanding when interactions between chemical and parasitic stressors take place, may help us in the dual purpose of fighting pests with minimized use of chemical pesticides, while ensuring a healthy environment for beneficial insects.

The aim of the present meta-analysis is therefore twofold: first, to provide a short overview of available reference models in ecology and (eco)toxicology with emphasis on features of the experimental design and model choice affecting the outcome of the meta-analysis. Secondly, to quantify the frequency of synergistic and antagonistic interactions between known anthropogenic chemical stressors and natural infections and, if possible, subsequently define the characteristics of synergistic and antagonistic combinations as well as develop hypotheses concerning a biological or biochemical mechanism behind the interactive effects. The meta-analysis focus on arthropods, which is the group of organisms where most data is available.

### Overview of reference models

#### Predicting combined effects of multiple stressors using models

Synergy and antagony are defined, respectively, as a significantly increased or decreased observed effect of a combination of two (or more) stressors, compared to the predicted effect of the specific combination. Hence, to identify a synergistic effect, one must be able to predict the effect of the non-synergistic joint effect of two or more stressors. To this purpose one must employ a reference model assuming no interaction, a null model^18,26^. Schäfer and Piggott (2018) provides a good overview of null models used in multiple stressor research across the fields of ecology and ecotoxicology, and the assumptions behind them. The models are summarized in Table 1 using the annotation of Schäfer and Piggott (2018), where the effect of a stressor is described as a function of stressor intensity (SI). For chemical and parasitic stress, the SI will be either the dose/concentration of the chemical, or a quantitative measure of parasite load. The adverse effect will, in most cases, be a sigmoid or possibly linear function of stressor intensity. Common for all the models is that they assume no interaction between stressors and that they predict the joint effect on one endpoint at one point in time.

**Table 1:** Overview of different null models for two stressors, A and B, and their most commonly used names within the ecological and (eco)toxicological literature.

Ecological model	(Eco)toxicological model	Model assumption	Null model equation for multiple stressor research	Required input data
Simple Addition	Effect summation	Linear f(SI) relationships, or highly negatively correlated stressors, where individuals sensitive to stressor A are completely tolerant to stressor B and visa versa	*f*_AB_(SI_A_,SI_B_) = *f*_A_(SI_A_) + *f*_B_(SI_B_)	Effects for A and B at a given SI
Multiplicative	Independent action (IA) Effect addition Response addition Response multiplication	Independently acting stressors affecting a binary endpoint as for example mortality. The joint effect is then either the product of the non-affected fraction, or the sum of the affected fraction subtracted the product of the affected fractions	*f*_AB_(SI_A_,SI_B_) = *f*_A_(SI_A_) + *f*_B_(SI_B_) −* f*_A_(SI_A_) *f*_B_(SI_B_) or *f*_AB_(SI_A_,SI_B_) = 1 − ((1 − *f*_A_(SI_A_)) * (1 − *f*_B_(SI_B_))	Effects for A and B at a given SI as a fraction of the untreated control
Dominance	–	One stressor dominates to a level that the remaining stressors have no additional effects in a mixture	*f*_AB_(SI_A_,SI_B_) = max(*f*_A_(SI_A_), *f*_B_(SI_B_))	Effects for A and B at a given SI
–	Concentration addition (CA) Dose addition	All stressors affect the same physiological process. Hence, SI can be added when all stressors are converted to the same SI-unit, and the joint effect can be predicted from the sum of relative SI using a common f(SI) relationship	*f*_AB_(SI_A_,SI_B_) = *f*_A_(SI_A_ + γSI_B_) with γ = ESIx_A_/ESIx_B_	*f*(SI) relationships for both A and B, and stressor intensities ESIx_A_ and ESIx_B_ that result in the same effect size x, where x is typically selected to be 50% effect

Model assumptions and required input data are provided together with the equations used to calculate the joint effect of non-interacting stressors using the terminology of Schäfer and Piggott (2018). *f*_AB_(SI_A_, SI_B_) gives the joint effect of non-interacting stressors A and B as a function of the individual stressor intensity of A (SI_A_) and B (SI_B_), respectively. For further detail see Schäfer and Piggott (2018)^18^. Some null models are commonly used in both ecology and (eco-)toxicology but identified under different terminologies. Hence, when relevant both terminologies are listed.

Biological systems are, however, dynamic in time, and stressor effects occur on many biological levels ranging from molecular to individual, population and community levels. Null models should therefore ideally be able to predict joint effects on several biological endpoints, and to include development of effects over time^21,22^. This has been attempted, for example, by combining dynamic energy budget theory with the above null models to account for multiple stressors on different endpoints such as growth, reproduction, and survival^27^. Further extensions to population levels are also being attempted^28^. The data required for parameterization of such models is, however, comprehensive and the studies are therefore still relatively rare. For the purpose of a meta-analysis of existing studies on the combined effect of chemical and parasites a simpler approach is therefore needed, and choices in terms of data requirements have to be made: first, we need an endpoint for which sufficient data can be derived. Secondly, we need a model that can be theoretically justified, and finally, we have to choose the most appropriate time point for assessing deviations from the null model for the studies where data on multiple time points are given. These three requirements are discussed in further detail below.

#### Choice of endpoint

Mortality is the most frequently reported effect measure across stressor combinations and species in studies combining chemical and parasitic stressors in arthropods, and is therefore the chosen endpoint of this meta-analysis. Most of the studies combining chemical and parasitic stressors represent parasites that are known to increase host mortality as part of their transmission between hosts (fungi and viruses) indicating that host mortality is an appropriate endpoint. However, other parasites rely on host survival to ensure their own transmission (e.g. helminths), and while their effects on host fitness and synergy with chemical stressors may still be present, they would be more subtle than what can be measured by host mortality alone. To those parasites, sublethal endpoints such as growth and reproduction would be important to include if results are to be used in population models, but require long terms studies and are therefore rarely given. The effects of parasitic stressors based on host mortality is therefore likely an underestimation of the true effects of the combined effects of parasites and chemical stress. Mortality in the untreated and uninfected controls should ideally be zero to ensure that the effects observed in the treatments are caused by the treatment and not by other stressors. This is, however, not always the case and treatment mortalities are therefore often corrected by control mortalities.

#### Choice of reference model

The choice of reference model will be guided by the endpoint monitored (binary or gradual) and the mode of action of the stressors (Assumptions in Table 1). The stress imposed by a chemical exposure and a parasitic infection, respectively, is likely to affect different physiological processes in an organism. Chemical stressors mainly act on specific molecular targets e.g. specific enzymes or receptors, and/or they will elicit an up-regulation of specific detoxifying enzymes such as cytochrome P450s, esterases or transferases^29^. Parasite infections likewise elicit an immune response, depending on the type of infection and the immune system of the targeted organism^30^. Both the inducible detoxification mechanisms and the induced immune responses are energy demanding processes, as are the repair processes following tissue damage from the two types of stress. Even though there might be physiological cross-talk between detoxification systems and immune responses^31^, or parasites may themselves release chemicals providing a similar response to pure chemical exposure^32^, for the purpose of choosing an appropriate null model, we will consider stress by chemicals and parasites to act independently of each other on the binary endpoint of mortality. We therefore choose the Multiplicative model, in this meta-analysis to follow the ecotoxicological terminology (Table 1). Note that the Multiplicative model does not describe physiological mechanisms of interactions between parasites and chemicals. Rather, it provides a tool to identify whether unexpected interactions take place and if there are no interactions a tool to predict effects of combined stressors such as parasitic stress and chemical stress on the mortality of host organisms.

**Figure 1:**
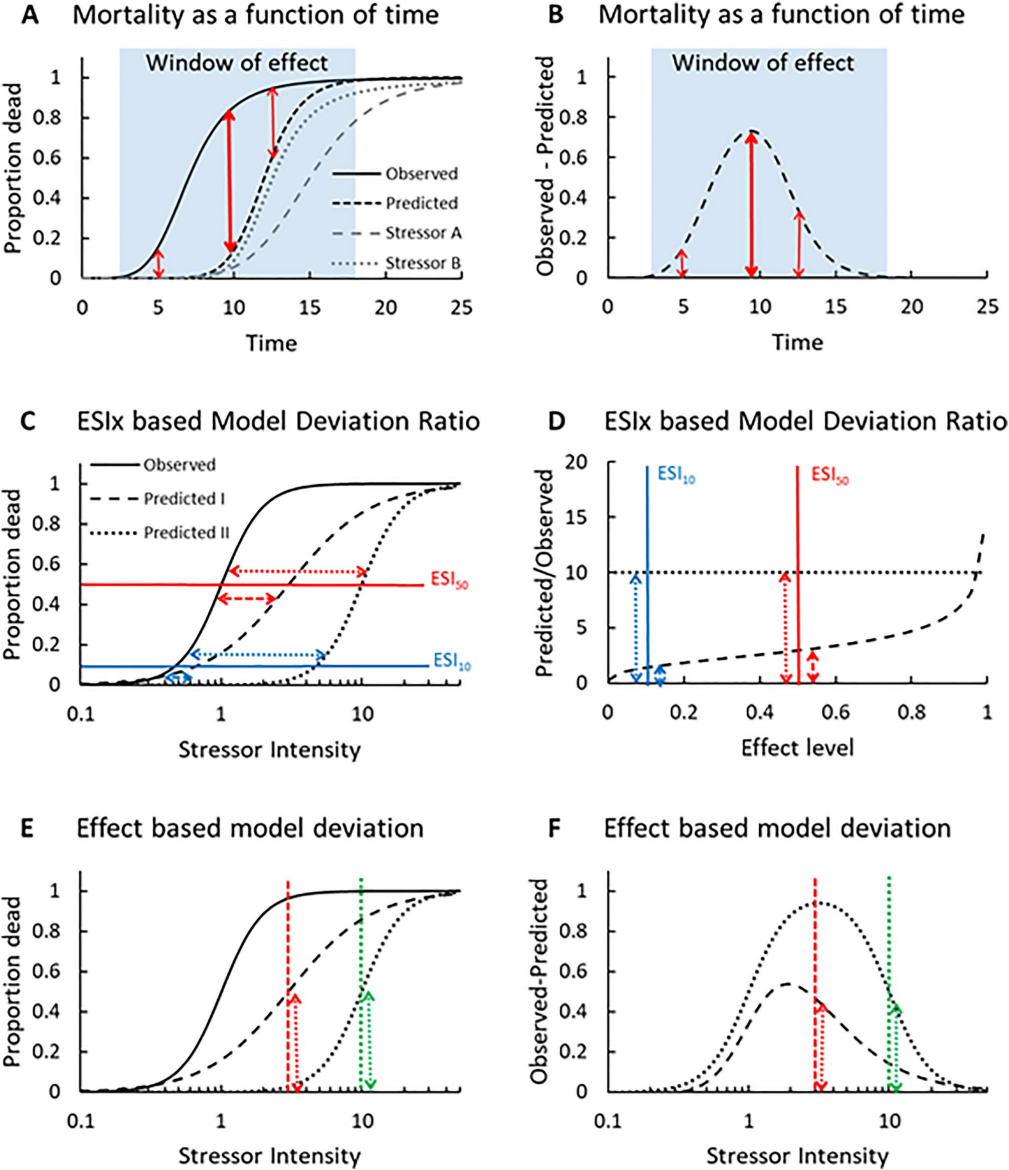
The mortality as a function of time of two individual stressors, their combination and the prediction using the Multiplicative model (**A**), and the difference between observed and predicted mortality as a function of time (**B**). Vertical arrows show the difference between the observed and predicted mortality at selected time points (thick arrow is the time-point of maximal difference). The blue area shows the time interval with differences between observed and predicted effects. Quantifying interactions using Concentration Addition as a null model and the ratio between predicted and observed effects (the model deviation ratio) at specific stressor intensities (ESI_x_) (**C,D**). (**C**) Observed mortality as a function of cumulated stressor intensity of a stressor combination together with two hypothetical predictions: one where the predicted curve is parallel to the observed curve (dotted curve) and one where the predicted curve has a lower slope than the observed curve (broken curve). Blue and red horizontal lines indicate the difference between observed and predicted curves at the two effect levels ESI_10_ and ESI_50_. The ratio between predicted and observed effects as a function of effect level (**D**), showing that the ratio between the two parallel curves is independent of effect level (dotted line), whereas it varies for non-parallel curves (broken line). For non-parallel curves, the median model deviation ratio is at ESI_50_. The two effect levels ESI_10_ and ESI_50_ from (**C**) are given in blue and red vertical lines, respectively, in (**D**). Quantifying interactions using Multiplicative as a null model at different stressor intensities (**E,F**). (**E**) Equivalent to (**C**), but when using IA as a null model the difference between observed and predicted curves are measured as the difference in mortality illustrated by the vertical red and green line crossing the ESI_50_ of the two predictions. (**F**) The difference between observed and predicted mortality (varying between 0 and 1) as a function of stressor intensity and illustrates that the difference between observed and predicted mortality is extremely dependent on the chosen stressor intensity.

The reason for not choosing the Simple Addition model (Table 1) is that the stressor intensity (SI)-effect relationships for the individual stressors, to our knowledge, are all sigmoid rather than linear, and the individual sensitivities are not consistently negatively correlated, as is requested of data to be used for Simple Addition^18^. In addition, just adding effects can lead to > 100% mortality, which is not biologically possible. Simple Addition underestimate the synergy compared to the Multiplicative model, as it only adds the effects without subtracting their product (Table 1). However, as it was used in a recent meta-analysis of agrochemicals, nutritional and/or parasitic stressor interactions^9^, we included it to quantify the difference in observed versus predicted results. The Dominance model will not be considered, as its applicability is restricted to plant science. The Dominance model assumes that the most limiting nutrient determines the growth rate of plants, irrespectively of the availability of the other nutrients^18^. In contrast to that assumption, when combining chemical and parasitic stress, none of the stressors renders the individual insensitive to the other stressor. Finally, full SI-effect relationships (Fig. 1C) are required to make mixture predictions using Concentration Addition^18,26^; and even though chemical and parasitic stress could be argued to act through a common mode of action, all leading to the death of the organism, SI-effect relationships are rarely included in studies of parasitic stressors. Hence, for the purpose of this meta-analysis, it cannot be used.

#### Choice of time point of assessment

Mortality as a function of any stressor is a process in time, hence, both the timing of the application of the individual stressors, and their sequence, will affect their final joint effect, as will the specific time when effect is measured^33,34^. Several studies monitor mortality as a function of time, which would ideally look as in Fig. 1A for stressor A, stressor B and their combination. If monitoring joint effects too early, no effect is found, and if monitoring too late, all organisms in one or more treatments may be dead (Fig. 1). Hence, it is important to choose the time of joint effect assessment in the time window of intermediate effects. However, even within this time-window, the difference between observed and predicted effects will vary in time, giving rise to different levels of synergy or antagony depending on when the interaction is observed (Fig. 1B).

### Quantifying interactions

Meta-analyses of effects of chemical mixtures most often employ Concentration Addition as the default null model. For the Concentration Addition model, the deviations between observed and predicted mixture effects are given as the ratio between observed and predicted effect concentrations, usually the 50% effect concentration (EC50)^12,14,15^ (Fig. 1C, D). The assessment answers the question: how much more or less chemical does it take to achieve the same toxicity from the mixture, compared to what was predicted from knowledge of the effect of the single chemicals? The advantage of comparing effect concentrations is that you can quantify infinitely large synergistic and antagonistic interactions. If your effect curves are parallel, as shown for the observed and predicted (II) effect in Fig. 1C, the quantified synergistic or antagonistic response will not be affected by the choice of effect level (Fig. 1D). Even for non-parallel curves, ratios between observed and predicted effect concentrations are reasonably stable within ECx values (or ESIx values, using stressor intensity (SI) as the independent variable) typically used for either risk assessment (EC10) or crop protection (EC90). The ratio between predicted and observed effect concentrations has been termed Model Deviation ratio (MDR) or synergy ratio^12,15^. MDR-values can also be calculated for combined stress predictions using the Multiplicative model as long as full SI-effect curves for the individual chemicals or stressors exist^35,36^. However, if full SI-effect curves are not available, this approach to quantify the difference between observed and predicted combined effects cannot be used. The alternative is to compare the difference between the observed and predicted effect at a specific combination of stressors and stressor intensity (Fig. 1E). This approach answers the question: how much extra mortality (or survival) occurs in the combined treatment, compared to what was expected from the effect of the individual treatments?

Contrary to stressor intensity (chemical concentration or parasite load or a combination of the two expressed as relative stressor intensity), which is in principle numerically infinite, the effect in terms of fraction of dead or survived individuals, will always vary numerically between zero and one. In addition, comparing effects at a specific stressor intensity, will give very different results depending on the choice of stressor intensity, contrary to when comparing effect concentrations at a specific effect level on approximately parallel stressor intensity-effect curves (Fig. 1C–F). The quantification of synergy or antagony based on effects will therefore be much more variable and stressor intensity dependent, compared to the more robust quantification of interactions based on stressor intensities yielding a specific effect. Using a ratio between observed and predicted effects also does not make sense when evaluating effects, as dividing with very small effects would make the ratio go towards infinity. Hence, for quantifying interactions on effect data it makes more sense to simply quantify the difference in mortality or survival by subtracting the observed with the predicted effect, and expressing this difference as an increase or decrease of the fraction of dead or survived individuals (Fig. 1F).

Thus, aiming at quantifying deviations from the Multiplicative model on combinations of chemical and parasitic stressors poses (at least) two challenges, which we are rarely faced with when quantifying interactive effects on chemical mixtures using Concentration Addition: first, at what point in time should a deviation between observed and predicted effects be quantified? This challenge also applies to chemical mixtures. However, as studies of effects of chemical mixtures usually apply fixed time-points based on standard experimental protocols (limiting in information as they may be), studies involving parasites more often make sequential observations of mortality over time. Hence, for the purpose of this meta-analysis, we had to choose the most relevant time point to quantify deviations between observed and predicted effects. We chose to use mortality data from the time point resulting in the highest deviation from the Multiplicative model in the studies where mortality for several time points were given. This was considered the most relevant data, since the larger reduction/increase in mortality during the lifetime of a population is likely to be the determining factor of the damage done to crops and/or growth of the population, than will the minor differences observed at other time points. The second challenge is to determine at what stressor intensity a deviation between observed and predicted effects should be quantified, if more stressor intensities were tested. According to Fig. 1E and D low stressor intensities should make it more likely to detect synergistic interactions, while high stressor intensities would make it more likely to detect antagonistic interactions. Hence, we chose to include all stressor intensities if more than one was tested, acknowledging the bias it might give to specific stressor combinations that were tested at different intensities, and then quantify the effect of stressor intensity on the degree of deviations from the reference model. Finally, common to both combinations of chemicals and of chemicals with parasitic stressors is that the ratio between the stressors and the sequence by which they are applied matter to the final joint effect. Thus, finding no synergistic or antagonistic effects in an experiment does not mean that it cannot occur for that stressor combination, if applied at another intensity, ratio or sequence. Likewise, finding an interaction does not mean that it will always happen if applied at another intensity, ratio or sequence. In addition, common for all meta-analyses is that the results will be biased in terms of what data are available. Often experiments are set up to find synergistic interactions, hence the dataset is likely biased towards synergy. Likewise, the choice of test organisms will be biased towards known pests for which synergistic interactions for eradication are sought, and towards sensitive pollinators whose recent decline we strive to understand. All the above should be kept in mind when interpreting the data.

## Results and discussion

### Composition of data

**Figure 2:**
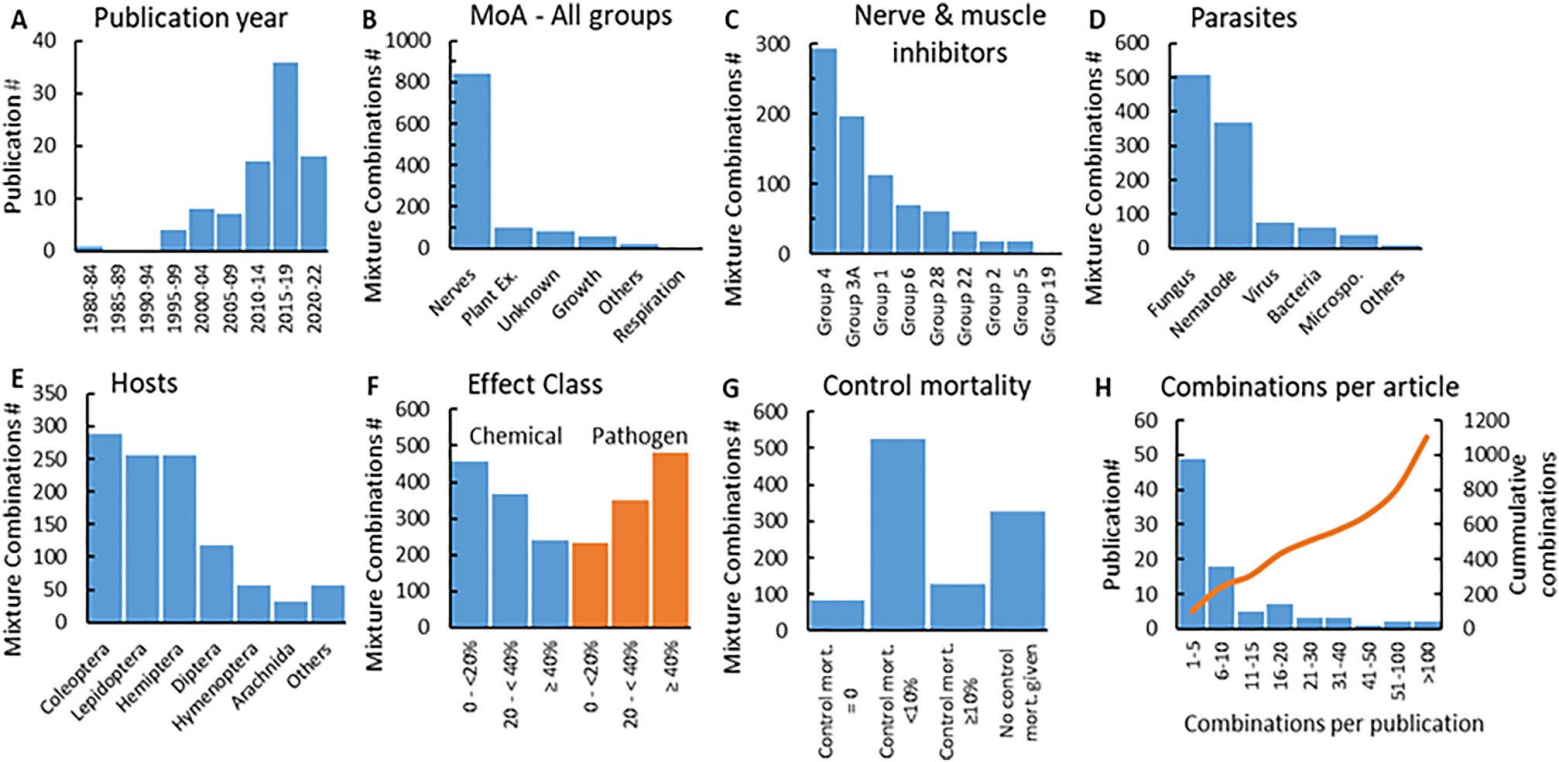
Number of chemical/parasite stressor combination studies as a function of publication year (**A**), the distribution of chemical mode of action (MoA) (**B,C**), parasite (**D**) and host phylogenetic class (**E**), effect size studied (**F**), the size of control mortality (**G**), and the number of combinations per publication (**H**). The number of publications passing the final criteria is given in intervals of five years, except for the last interval, which only includes 2½ year (**A**). The overall MoA, based on the IRAC classification^38^: nerve and muscle inhibitors (Nerves), growth inhibitors (Growth), respiration inhibitors (Respiration), unknown MoA (Unknown) and plant extracts (Plant Ex) (**B**). The category “others” is a mix of repellants, a fungicide and chemicals not categorized by IRAC. Due to the large number of “nerve and muscle inhibitors” this MoA group was, split into smaller subgroups according to IRAC (**C**). The group numbers reflect the following insecticide MoA, followed by the subgroups or compounds tested in parentheses: Group 4: nicotinic acetylcholine receptor competitive modulators (neonicotenoids, nicotine, butenolides and sulfoximines), group 3A: sodium channel modulators (pyrethroids), group 1: acethylcholine esterase inhibitors (organophosphates and carbamates), group 6: glutamate-gated chloride channel alosteric modulators (avermectins), group 28: ryanodine receptor modulators (diamides), group 22: voltage-dependent sodium channel blockers (semicarbazones), group 2: GABA-gated chloride channel blockers (fibronil), group 5: nicotinic acetylcholine receptor allosteric modulators (spinosad), group 19: octopamine receptor agonists (amitraz). The parasites were divided according to phylogenetic groups: Fungi, nematodes, viruses, bacteria, microsporidia (“Microspo.”) and “others” encompassing mites, and protozoa (**D**). Hosts were mainly: Coleoptera (beetles), Lepidoptera (butterflies & moths), Hemiptera (aphids, cicadas and other plant sucking insects), Diptera (mosquitoes and flies) and Hymenoptera (bees). Three species of the class Arachnida were also tested together with a few crustacean species, the latter being allocated to the group “others” together with insects of rarely tested orders (**E**). The level of control mortality and individual stressors affected the results, and hence the frequency of combinations within different effect classes are given (**F,G**). Finally, the number of combinations from each study was provided with the cumulated number of combinations for each group of studies (**H**).

The available publications strongly affect the composition of the database (Fig. 2). The figure verifies the increased interest in chemical and parasite combinations over the past 40 years, showing only one study during the first fifteen years (1980–1994) to 36 publications in the last full five-year interval (2015–2019) (Fig. 2A). In most publications, tested chemicals are insecticides targeting nervous system functionality, with neonicotenoids, pyrethoids, organophosphates, and carbamates being part of approximately 60% of the 1064 stressor combinations (Fig. 2B, C). Fungi and nematodes were included in > 80% of the combinations, and the most prevalent hosts were larvae or adults of beetles, butterflies and moths, which together with nymphs of plant sucking insects were used in > 75% of the combinations (Fig. 2D, E). The focus on these host species, which all act as agricultural pests, show a strong bias towards publications investigating chemical and parasite combinations with the likely aim of decreasing the use of conventional insecticides by combining them with more naturally occurring parasites. Including the group of publications conducted on mosquitoes and flies as examples of publications targeting insect pests, the proportion of combinations on pests increase to 86%, leaving combinations on non-target species such as bees, crustaceans and more rare insect classes (of which some are also pests) to 14%.

Evaluating the stressor levels typically used in the publications, the chemical stressors were tested almost twice as often at low effect levels (0- < 20%) compared to high effect levels (≥ 40%), whereas for parasites high effect levels dominated the dataset (Fig. 2F). This most likely reflects the study aim of the majority of the studies being to test to what degree the effect of a biological control agent (parasite) can be enhanced by adding small amounts of an insecticide. Studies on non-target organisms are likely to test if sub-lethal or low mortality exposures to insecticides enhance host susceptibility to low doses of parasites, which also favors low insecticide doses. Of the 739 combinations where control mortality was given, it was < 10% in 82% of the cases signifying good quality studies in the majority of the combinations (Fig. 2G). The last graph (Fig. 2H) shows that 49 of the 90 scientific publications contributed with less than 5 stressor combinations each, jointly contributing with approximately 10% of the combinations. In contrast, only eight publications contributed with approximately half the combinations with the top four contributing with 41%. This gives the dataset a very strong bias towards the chemicals, parasites and hosts used in these four publications^41–44^.

### Main results and methodological issues influencing deviations from predictions

#### Synergistic effects dominate

**Figure 3:**
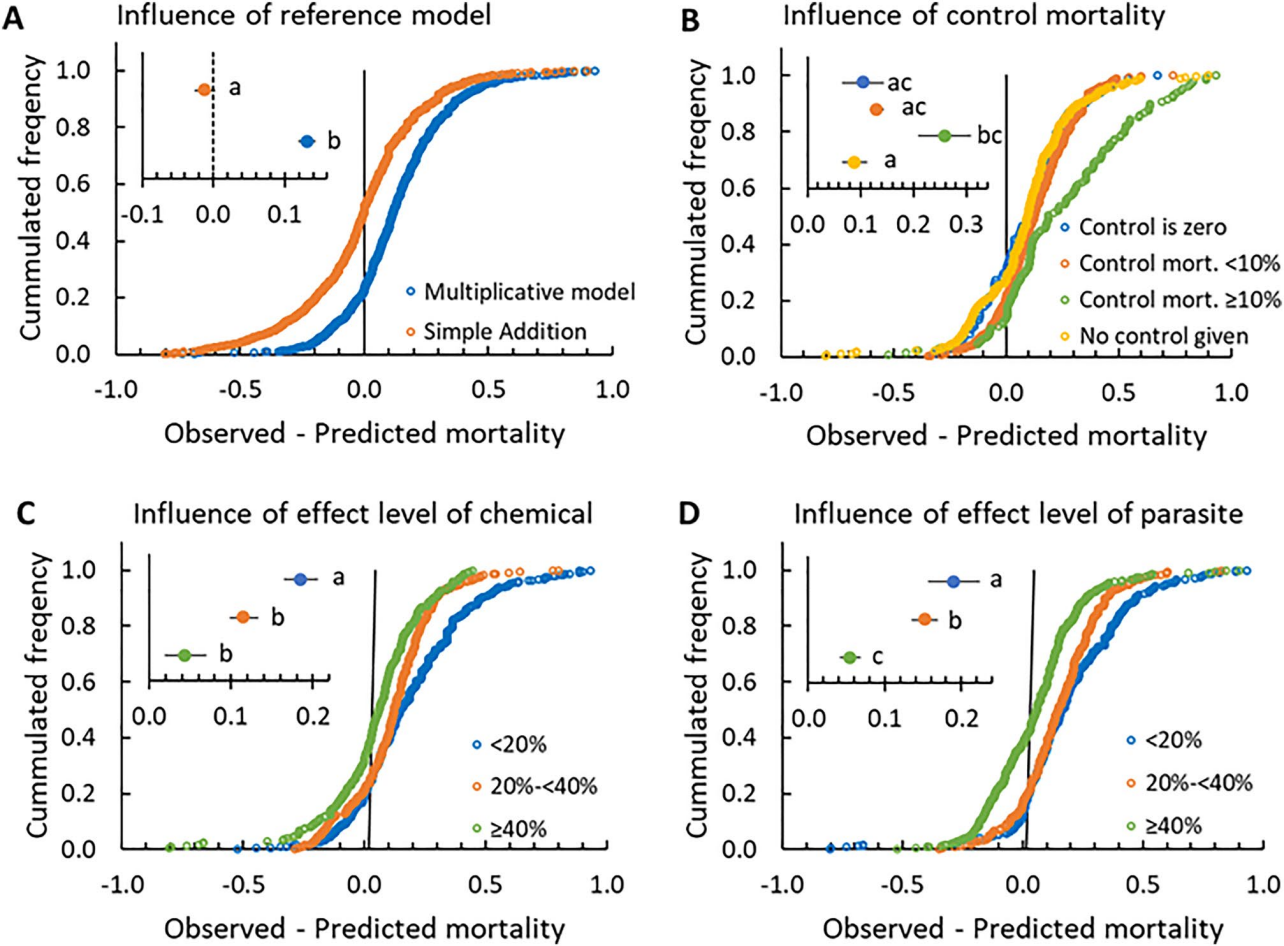
The cumulative frequency of stressor combinations as a function of the difference between observed and predicted effect of the stressor combination. Negative values indicate antagonistic effects while positive values indicate synergistic effects. Inserts give mean ± 95% confidence intervals, with significant differences between treatments annotated by different letters (linear mixed model with publication as random effect, followed by a Tukey test, *α* = 0.05). The panels show the consequence of model choice (**A**), of the level of control mortality (**B**), and of the effect level included in the stressor combinations for either the chemical (**C**) or the parasite (**D**).

The vast majority (77%) of the combinations of chemical and parasitic stressors showed synergistic interactions with an average excess mortality of around 13% relative to predicted mortality (distribution mean ± 95%CI: 0.131 ± 0.013, Fig. 3A). This result is in strong contrast to meta-analyses on chemical mixtures, where only a relatively small percentage (< 10%) of the combinations are synergistic^12,14,15^. The exception are recent meta-analyses focusing on pesticide mixtures on bees, which are almost 100% synergistic^9,13^. This is due to most of the tested mixtures in bee-studies include inhibitors of the main biotransformation enzymes: P450 monooxygenases (pipenoryl butoxide and azole fungicides) or esterases (some organophosphates), known to induce synergy in combination with chemicals whose biotransformation depends on esterases^9,12,13,45,46^*.* A review of meta-analyses of stressor interactions in ecology by Côté et al. (2016) found synergistic interactions in 4–68% of the respective meta-analyses depending on species or communities investigated, measured endpoints and, not the least, the null models used^20^. The frequency of 77% synergy in the present study should be interpreted with caution, as it is simply the proportion of studies with a positive observed minus predicted value. There are uncertainties both on the input data for the model and on the observations, and if these are accounted for, the frequency of statistically significant synergy would be considerably smaller. For chemical mixtures using Concentration Addition, data has shown that deviations from the reference model of less than two-fold are rarely experimentally reproducible^47^. Hence, synergy and antagony are often only considered biologically significant if there is more than a two-fold deviation between observed and predicted using Concentration Addition as the null model^12,14^. Similarly, experimentally based uncertainties on mortalities in combination studies are rare to find, but assuming that combinations within a range of ± 5% of the predicted value are not deviating from predictions, 66% of the combinations in the present study would still be synergistic. More focus on statistical analysis of deviations and ecological variability within and between studies is, however, needed to be able to distinguish true statistical and biological interactions from random experimental deviations^19^.

#### Control mortality matters

For the observed synergies between chemical and parasites in this study, it could be hypothesized that the synergistic interactions are a result of competition between energy demanding processes. The upregulation of physiological processes related to detoxification or damage repair typically occurring at sublethal doses/concentrations of chemicals are likely to use energy reserves that could otherwise be spent on parasite avoidance behavior (i.e. grooming to remove infective fungal spores) and upregulation of an immune response targeting parasites,- and visa versa. An indication that competition for energetic reserves can result in synergy was presented in a recent meta-analysis on combined stressors on bee mortality including nutrition stressors in addition to agrochemicals and parasitic stress^9^. In this study, the highest frequency of synergy between different stressors was found in combinations of parasitic and nutritional stress^9^. Additional stress on top of chemical and parasitic stress might also be the cause of the significantly higher level of synergy in combinations where control mortality was ≥ 10% compared to combinations with no or low (< 10%) control mortality (Fig. 3B). High control mortalities are a sign of the organisms not thriving in the test setup, and the meta-analysis results clearly show this to be an interfering factor for the quantification of the interactions between two specific stressors. In nature, organisms are likely to experience more than two stressors, hence, the effects of multiple stressors under natural living conditions may well be more severe than those observed in the lab, where test organisms are ideally well fed and thriving.

#### Stressor intensity matters

In the theoretical section it was hypothesised that it is more likely to detect synergistic interactions if using low stressor effect levels, and similarly more likely to detect antagony using higher effect levels. This is simply due to the fact that using mortality as an endpoint and the Multiplicative model as the reference model, the lower and upper limits for effects are set to zero and 100% (Fig. 1). The theoretical assumption was confirmed by data as combinations using low effect levels of chemical and parasitic stressors (< 20%) showed significantly higher levels of synergy than combinations with higher effect levels (Fig. 3C, D). Using effect levels > 40% increased the proportion of antagonistic combinations for both stressors, but only significantly so for the parasitic stressor. The limitations of the Multiplicative model in terms of fixed upper and lower limits could be alleviated by using an approach similar to that of Concentration Addition (Fig. 1C, D; Table 1). That would require full stressor-effect relationships for all stressors included, but would enable effect prediction using the model framework that has shown to explain the vast majority of chemical mixtures^12,14,15^. Hence, it might also be a superior prediction model for more diverse types of stressors. That said, comparisons of observed and predicted chemical mixture data did not show a very big difference in the predictive ability of Concentration Addition and the Multiplicative model^36^, but whether that also applies to other stressor combinations remains to be tested.

#### The time component

The stressor effect bias may also be a consequence of using mortality data at one fixed point in time. Figure 1A, B, clearly show that the magnitude of deviation, and possibly the type of deviation could depend severely on the observation time. High stressor levels would be low stressor levels, if observed at an earlier time point, while low stressor level may or may not increase at later observational times, depending on when data are collected relative to when the stressor effect is shown. Hence, ignoring the effect of time as done in this meta-analysis, due to lack of consistent time-resolved data, may be the largest source of systematic error. Knowing that stressor sequence and exposure intensity will also vary in time in a more natural setting^21^ emphasizes the use of models that include stressor intensities and effects in time. The toxicokinetic and toxicodynamic dynamic models used in ecotoxicology combined with population and food-web models used in ecology will be necessary to describe and predict combined stressor effects, as has been concluded in a number of studies and meta analyses on multiple stressors; particularly if integrating different biological levels of effects^20–22^. Using dynamic models, the applicability of different null models can also be tested, as for example described by Bart et al. (2022) for the very simple TKTD model GUTS^48^.

#### Choice of null model

The Concentration Addition (Table 1) model could not be tested on the data collected due to the lack of SI-effect relationships in most studies. The comparison between the Multiplicative model and that of Simple Addition/Effect summation was conducted despite of the fact that Simple Addition assumes that more than 100% mortality is possible, as it has been used in other meta-analyses^9,49^. The comparison showed, as expected from the mathematical derivations of the equations, that Simple Addition predicted less synergy moving the distribution into the area of additivity (mean ± 95%CI: -0.014 ± 0.017, Fig. 3A). The only other meta-analysis we could find testing both models on the combined effects of insect pests and plant diseases on plant health also found less synergy (4%) using simple addition compared to using the Multiplicative model (9%)^49^. Hence, unless the stressors fulfill the assumptions of having a linear relationship between stressor intensity and the measured endpoint (which neither chemicals nor those parasitic stressors we have seen tested in full intensity effect relationships do), or unless being highly negatively correlated stressors^18^, the Multiplicative model is the only correct model to use for chemical-parasite stressor relationships evaluated on binary endpoints such as mortality.

### Biological causes of synergistic interactions

#### Effect of chemical stressor

**Figure 4:**
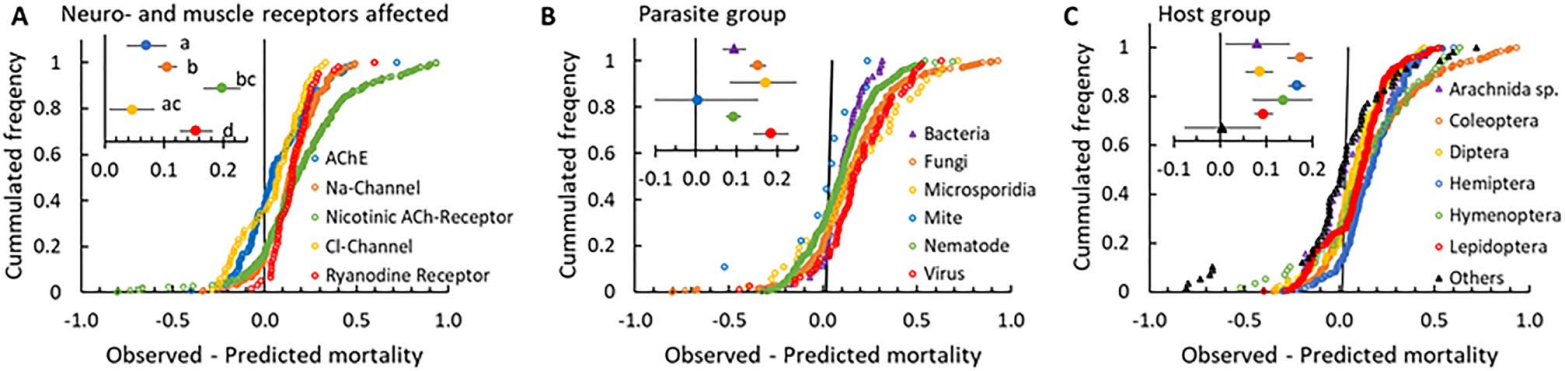
The cumulative frequency of stressor combinations as a function of the difference between observed and predicted effect of the combinations for the studies divided into groups depending on the mode of action of the tested chemicals (**A**), or the taxonomy of the parasite (**B**) and the host (**C**). Negative values indicate antagonistic effects while positive values indicate synergistic effects. Inserts give mean ± 95% confidence intervals, with significant differences between treatments annotated by different letters (linear mixed model with publication as random effect, followed by a Tukey test, *α* = 0.05).

The chemical stressors grouped into 19 IRAC MoA categories for insecticides (83% of all combinations), plant extracts (9%), antioxidants (0.4%), repellents (0.2%), SDHI fungicides (0.1%), and a group of compounds with unknown mode of action (8%). To test if the way the chemical affect host physiology can affect the outcome of the chemical-parasite combination, we chose to focus on the IRAC classified chemicals with > 10 combinations per class. This reduced the groups to 9 classes (IRAC class: 1–7, 22 and 28) of which all but one affect the signaling processes in nerves or muscles. The exception was pyriproxyfen belonging to group 7 (*n* = 26), which is a juvenile hormone mimic. To further reduce the groups and increase the number of combinations per class, fibronil (class 2, *n* = 18) and avermectins (class 6, *n* = 71) were grouped together, as they both affect chloride channels; spinosyns (class 5, *n* = 18) were grouped with the neonicotenoids (class 4, *n* = 293) due to their common effect on the nicotinic acethylcholine receptors, and indoxacarb and metaflumizone (class 22, *n* = 32) were included in the group of pyrethroids (class 3, *n* = 197) as they all affect sodium channels. The results showed combinations including acethylcoline esterase (AChE) inhibitors, Na-channel and Cl-channel inhibitors to have the lowest average level of synergy, and nicotinic ACh-receptor inhibitors, ryanodine receptor inhibitors and juvenile hormone mimics to have on average higher levels of synergy (Fig. 4A). Due to combinations from the same publication being considered less independent than those from different publications, the test for significance grouped the MoA slightly differently with combinations including AChE and Cl-channel inhibitors being significantly lower than the Nicotinic Ach-receptor and ryanodine receptor inhibitors. Considering only the three specific classes with most combinations (AChE inhibitors, pyrethroids and neonicotenoids with *n* = 113, 197 and 293, respectively), AChE inhibitors were significantly less synergistic than both pyrethroids and neonicotenoids (*p* < 0.001), while there were no significant differences between the latter (*p* = 0.64).

Above, we hypothesized that synergy between chemical and parasitic stressors could occur due to competition between energy consuming processes. The question is if organophosphates and carbamates demand less energy for detoxification or damage repair compared to pyrethroids and neonicotenoids? There is, to our knowledge, no indications that detoxification costs are different between the different insecticide groups, which are all biotransformed by a wide range of cytochrome P450 and esterase catalyzed processes. However, the cost and time-course of damage repair could be different. Organophosphates and carbamates (AChE inhibitors) both inhibit the enzyme acetylcholinesterase^38^, while the pyrethroids bind irreversibly to the voltage-gated sodium channels in the nerve cells preventing their closure (and possibly also to other ion channels)^38,50^, while neonicotinoids bind irreversibly to the nicotinic acetylcholine receptors^38^. It could be hypothesized that substituting damaged enzymes as acetylcholinesterase (AChE) is a relatively quick and energetically cheap repair process, as these enzymes are continuously renewed, compared to the renewal of ion channels or nicotinic acetylcholine receptors, which are both larger and more complex membrane embedded entities consisting of several subunits^51^. This hypothesis was, however, not confirmed by the 70 combinations including Cl-channel inhibitors, where synergy was as low as for the AChE inhibitors.

The difference in level of interaction between the combinations including the three largest groups of insecticides could also be due to a bias linked to the parasite or species used in the combination, or to methodological issues such as the size of control mortality or effect level of the chemical or parasitic stressor used. The combinations showed no skewed distribution relative to the above factors affecting synergy in the AChE-inhibitor combinations relative to either the pyrethroids or neonicotenoids (Table S1), with two exceptions: (1) The frequency of combinations with control mortality ≥ 10% was higher in the neonicotinoid combinations compared to the AChE-inhibitor and pyrethroid combinations (36% versus 11% and 4%, respectively), and (2) the frequency of low effect levels of parasitic stress was higher in the neonicotinoid combinations compared to the AChE-inhibitor and pyrethroid combinations (34% versus 16% and 14%, respectively). Testing the difference in synergy omitting data with control mortalities ≥ 10% or parasite effect levels < 20%, however, gave the exact same results. Hence, the difference in synergistic potential between the chemicals is likely related to their MoA, though the specific causal factors need to be identified.

#### Effect of parasitic stressor

Comparing the large groups (> 10 combinations) of tested parasites: fungi, nematodes, viruses, bacteria, microsporidia (*n* = 509, 369, 77, 61, and 39, respectively), there was no significant difference in their ability to cause synergy (Fig. 4B), though there was a tendency for combinations including viruses to be most synergistic, and those with bacteria and fungi least synergistic. As the parasite phyla might not be the most important trait in an infection that will affect an interaction with a chemical, several parasite specific characteristics were tested for influence on the deviation between observed and predicted mortality (Fig S1). None of the traits affected the output of the combination significantly, though parasites inducing the immune pathway JAK/STAT (mainly viruses, *n* = 79) seemed to induce slightly more synergistic interactions (*p* = 0.68), while parasites that did not reproduce in the host, or did not induce a mortal effect (n = 12 and 30, respectively) were less likely to be synergistic (*p* = 0.50 and 0.20, respectively)(Fig S1A, B, D). Parasites that infected local tissue (n = 40), the effect of transmission route (*n* = 181 and 872 for oral and topical transmission route, excluding the ectoparasites) and the production of secondary metabolites (SM) by the parasite (*n* = 186 and 879 for those that did not and did produce SM), also did not affect the outcome of the combination (*p* = 0.15, 0.31, 0.83)(Fig S1C, E, F).

Common for all the traits tested were that only a very small proportion of studies included parasites that did not reproduce in the host, were local or co-existing (*n* = 12, 40 and 30 out of the total of 1064 combinations). These are all traits that could be hypothesized would cause less stress and therefore less synergy than systemic parasites reproducing in and killing the host. Hence, to test hypotheses related to the influence of parasitic life-history strategy on synergistic interactions, more balanced datasets are needed ideally conducted on similar hosts and using similar stressor effect levels.

#### Effect of the host

Considering the host taxonomy and host characteristics, we did not have any prior hypotheses in terms of any phyla being more or less likely to host synergistic interactions than others. Comparing the main phylogenetics groups also showed no significant influence of the host on the outcome of the chemical-parasite combination (Fig. 4C).

## Conclusion and outlook

The present meta-analysis confirms our hypothesis that synergistic interactions, defined as higher mortality than predicted by the Multiplicative model, are more frequent than antagonistic interactions in studies combining chemical and parasitic stressors. The finding that experimental design and choice of data treatment methods play a larger role for finding synergy in > 1000 chemical-parasite combinations than biological factors do, however, emphasize the need for establishing guidance in terms of how to design mixed stressor studies across scientific disciplines. Testing single stressors alone (Stressor A at dose 1 and Stressor B at dose 2) and in combinations (Stressor A at dose 1 with Stressor B at dose 2), as is the case in for the most of the reviewed studies, may give information on the joint effect of exactly the tested case. These data, however, cannot be used to say anything general about the stressor interactions in any other scenarios than the tested, nor do they allow parameterizing predictive models, which could be used to foresee joint effects of non-tested stressor combinations. Including stressor intensity-effect relationships for individual stressors and time resolved measures of effects would be a good starting point to allow mechanistic insight in interactions across stressor combinations. For chemical-parasitic stressors especially, more systematic work using chemical and parasitic stressors, initiating different avoidance, coping (upregulation of defense mechanisms) and repair processes in different hosts are needed to test biology-based hypotheses behind the tendencies found for synergistic interactions in this study.

## Materials and methods

### Identification of relevant publications and stress combinations

**Figure 5:**
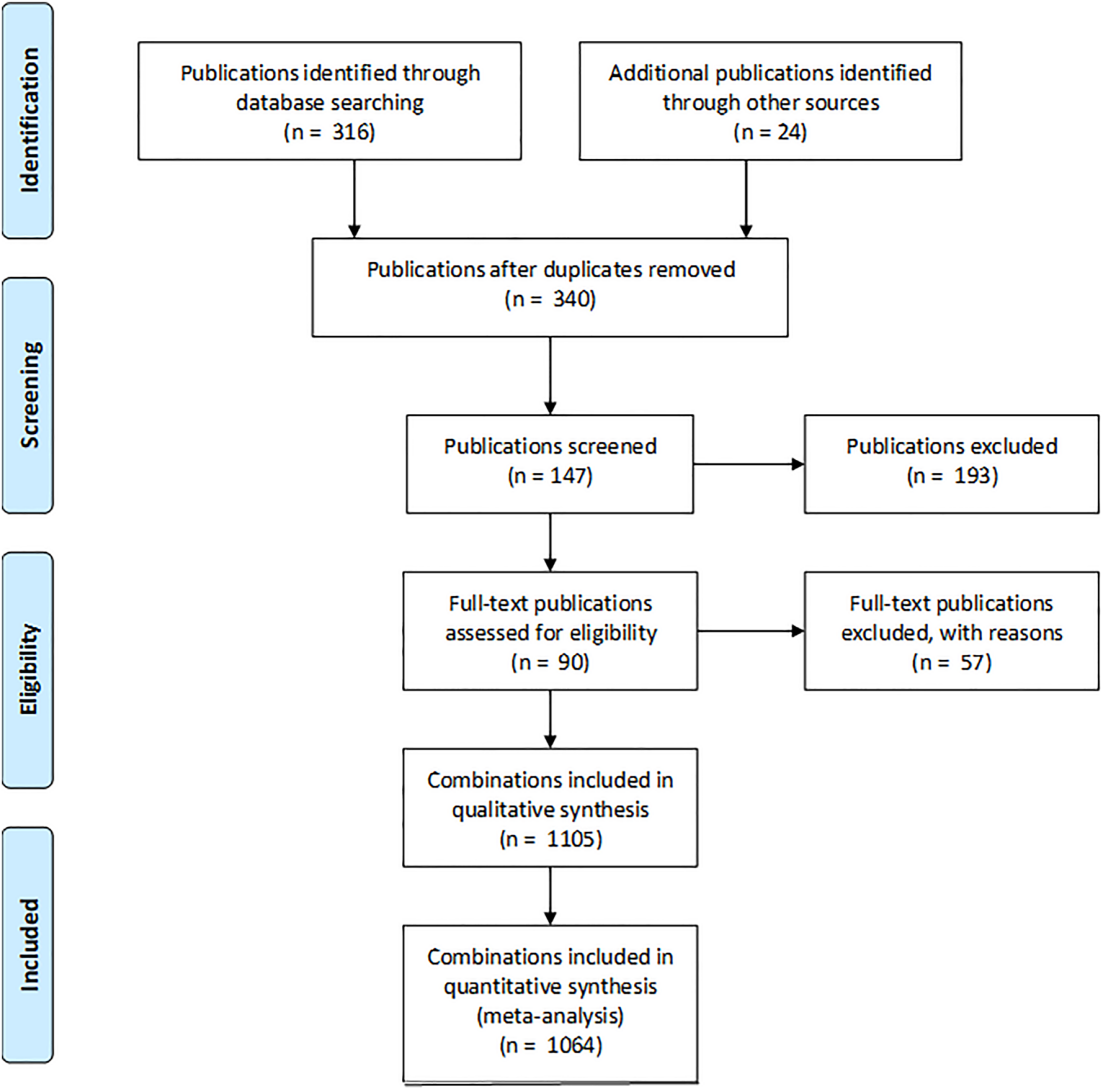
PRISMA 2009 flow diagram^37^. A flow diagram depicting the process of selection of records used in the review. Search criteria and criteria for selecting eligible records are given in the “Material and methods” section.

The systematic review was performed according to the PRISMA 2009 Checklist^37^. The flowchart in Fig. 5 illustrates the process of literature search, screening and final selection of relevant publications. We used the SIS Web of Science database using the following search terms: “TS = (parasite* OR pathogen* OR bacte* OR fungi OR fungus OR virus* OR vira*) AND TS = (pesticide* OR insecticide* OR chemical*) AND TS = (co-exposure* OR simultan* + expos* OR co-stress* OR combin* + exposure* OR combin* + treatment OR combin* + stress* OR multiple + stress* OR parasite-insecticide + interaction* OR parasite-pesticide + interaction* OR pathogen-insecticide + interaction* OR pathogen-pesticide + interaction* OR insecticide-parasite + interaction* OR pesticide-parasite + interaction* OR insecticide-pathogen + interaction* OR pesticide-pathogen + interaction* OR combin* + effect* OR synerg* + interact* OR antagon* + interact* OR interact* + effect*) AND TS = (arthropod* OR insect* OR pollinator*) NOT TS = (amphi*)”. The search was selected to capture the largest range of publications investigating the effects of combinations of parasites with chemicals.

The search was performed on April 27th, 2022 and resulted in 316 publications, which were joined with 24 publications found through other sources, giving a total of 340 publications. The 340 publications were subjected to an initial screening, where a total of 193 publications were excluded based on the following inclusion criteria: (1) the publication must be available in full text (5 excluded). (2) Data must be presented in a research paper, not in a review or meta-study (12 excluded). (3) Host organism must be arthropods (29 excluded). (4) Co-exposure to chemical and parasitic stressors must occur (124 excluded). (5) The parasite and not the isolated toxin (e.g. Bt toxin) must be used (7 excluded). (6) Stressors must not act as commensalist (1 excluded). (7) The chemical must be an organic chemical, and not a metal or mechanical stressor as e.g. diatomaceous earth (15 excluded).

From the set of included publications (147) only data from experiments complying with the following criteria were extracted: (A) The effect measured must be host mortality (fraction of dead and/or alive host organism). (B) Effects should be measured for both parasitic stressor and chemical stressor alone and in combination. (C) The stress level, exposure type and duration of the combined exposure to stressors should be identical to those tested on the individual stressors. (D) The effect of single and combined stressors should be assessed on the same day post stress application. (E) Host organisms should be experimentally infected with a parasite or a natural infection should be quantified.

These criteria excluded another 57 publications, leaving 90 publications from which combinations of stressors could be retrieved. If mortality post stress application was reported at several time points for the same experimental setup, mortality data from the time point resulting in the highest deviation from the null model was reported. If multiple stressor intensities, stressor ratios and/or exposure sequences were tested in the same study, these were all included as individual stressor combinations. This resulted in a final set of 1105 stressor combinations. For 40 of these combinations one of the two individual stressors induced 100% mortality, hence their combination also induced 100% mortality. In addition, one combination showed no excess mortality relative to the control. These combinations were considered outside the “window-of-effect” (Fig. 1A, B) and were therefore removed from the meta-analysis. This left 1064 combinations for the meta-analysis from which mortality for individual stressors and their combination were collected. In addition, if given, control mortality was collected.

For each combination, the following information relevant for the biological action of the stressors was included in the database as potential explanatory descriptors of stressor interactions: (1) active ingredient of the applied chemical, if available. For the large range of miscellaneous plant extracts used, they were denoted “Plant extracts”. (2) Mode of Action (MoA) of the chemical, if known. The majority of the chemicals were insecticides and hence the International Resistance Action Committee (IRAC)-MoA classification was used^38^. (3) Scientific name of the host and its phylogenetic class and order. (4) Scientific name of the parasite and its phylogenetic group: Bacteria, Fungi (Ascomycota), Microsporidia, Nematoda (including Nematode-bacteria consortia), Viruses, and others (arthropods and protozoa). (5) Descriptors for the parasite infection strategy and host response, which could be hypothesized to affect their interactions with chemical stress: (A) main immune pathway induced, (B) Does the parasite reproduce in the host tissue? (yes/no), (C) Does it have systemic or local effect? (D) Is the transmission route oral or topical? (E) Is the infection strategy lethal to the test organism? (yes/no), (F) Is the parasite known to produce secondary metabolites? (yes/no). In terms of the infection strategy being lethal or not, all parasitic infections can lead to a level of stress that is lethal. However, we distinguish between parasites where their life-history strategy is dependent on the lethality of the host, which is the case for all fungal pathogens and nematodes in this study, which all utilise the arthropod corpse for their proliferation. This is contrary to microsporidian parasites, which can fulfill their lifecycle in the gut of their hosts without necessarily killing them^39^.

The entire database are given as Supplementary material.

### Treatment of data

Combined effects were predicted using the Multiplicative model and Simple Addition/Effect summation (Table 1). Deviations between observed and predicted effects were quantified as the difference in the fraction of dead hosts at a specific stressor combination and intensity.

Control mortality was given for 739 of the combinations of which 84 had zero control mortality, while for 325 combinations control mortality was not given or “accounted for” in the original publication. For the 655 combinations with control mortality given (C_m_), it was subtracted from the measured mortality of the treatment (T_m_), and all data was made relative to the control (1 − C_m_) thereby giving the true treatment mortality (T_t_):

1 $${\text{T}}_{{\text{t}}} = \, \left( {{\text{T}}_{{\text{m}}} - {\text{ C}}_{{\text{m}}} } \right)/\left( {{1} - {\text{ C}}_{{\text{m}}} } \right)$$

However, in 42 and 37 the chemical or parasite treatments, respectively, control mortality was larger than treatment mortality (a total of 67 combinations was affected by one of the stress treatments having lower mortality than the control). This could either mean that the treatment had a beneficiary effect on mortality (or postponed mortality) or it could be a random effect. We cannot rule out the possibility that small doses of chemical or parasitic pressure can affect mortality by for example delaying mortality at the cost of reproduction, as has been observed for both chemical and parasitic stress^40^. However, for this meta-analysis we will consider it random. Hence, if control mortality is larger than treatment mortality, adjusted treatment mortalities are set to zero.

In addition to the biological descriptors used as explanatory factors for the degree of synergy or antagony, the following methodological descriptors were also included: (1) choice of reference model (IA versus Simple Addition), (2) level of control mortality and (3) effect level of the individual chemical and parasitic stressors.

The importance of different explanatory factors for the degree of synergy or antagony of a combinations was tested using a linear mixed model with the publication as random effect, followed by a Tukey test. Data treatment was performed in Excel and in R 4.0.0.

### Supplementary Information

Supplementary Information 1.

Supplementary Information 2.

## Data Availability

All data generated or analysed during this study are included in this published article [and its supplementary information files].
