# Supplementary material for: Quantifying synergistic interactions: a meta-analysis of joint effects of chemical and parasitic stressors
Source: Sci Rep. 2023 Aug 22;13:13641. doi: 10.1038/s41598-023-40847-6 (PMC10444819; doi:10.1038/s41598-023-40847-6)
Supplement: Supplementary file 2 — Supplementary Information 2. [file 41598_2023_40847_MOESM2_ESM.docx]

**Supplementary Material**

**Figure S1**


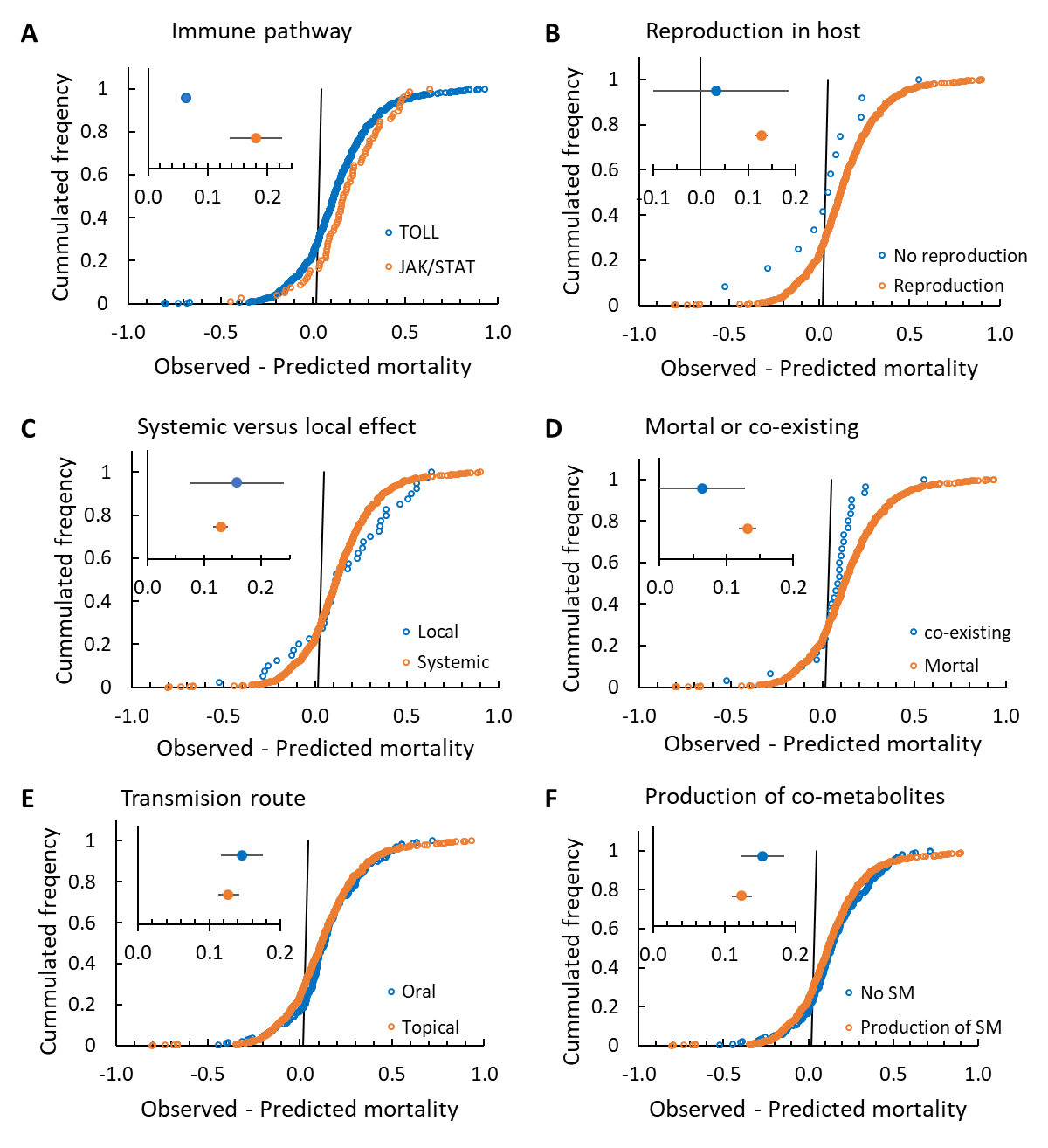


**Figure S1.** The cumulative frequency of stressor combinations as a function of the difference between observed and predicted effect of the mixtures divided into groups depending on features related to the parasite. Negative values indicate antagonistic effects while positive values indicate synergistic effects. Inserts give mean±95% confidence intervals, with significant differences between treatments annotated by different letters (Linear mixed model with publication as random effect, followed by a Tukey test, *α* = 0.05). The panels show the consequence of Immune pathway (A), Reproduction in the host (B), whether the parasite has systemic versus local effects (C), if it requires host mortality or not to complete its life-cycle (D), its transmission route (E) and whether it is known to produce co-metabolites or not (F).

**Table S1.** Frequency (%) of other factors that might have affected the difference between the occurrence of synergy in combinations with AChE inhibitors, pyrethroids and neonicotenoids (*n* = 113, 197 and 293). Significant factors, defined as contributing with >20% for one of the chemicals, where there was a higher than two-fold difference between chemicals are given in bold.

| **Interfering factors** | **AChE-inhibitor** | **Pyrethroid** | **Neonicotenoid** |
| --- | --- | --- | --- |
| **Parasite group** |  |  |  |
| - Bacteria | 12.4% | 1.0% | 2.1% |
| - Fungi | 31.9% | 49.7% | 63.2% |
| - Microsporidium | 5.3% | 1.0% | 5.2% |
| - Nematode | ***46.9%*** | ***47.2%*** | ***21.0%*** |
| - Virus | 3.5% | 0.5% | 4.8% |
| - Other | 0.0% | 0.5% | 3.8% |
|  |  |  |  |
| **Host group** |  |  |  |
| - Coleoptera | 36.3% | 52.8% | 34.0% |
| - Diptera | 0.0% | 8.6% | 17.2% |
| - Hemiptera | 21.2% | 24.4% | 24.7% |
| - Hymenoptera | 0.9% | 1.0% | 14.1% |
| - Lepidoptera | 29.2% | 12.2% | 10.0% |
| - Other | 12.4% | 1.0% | 0.0% |
|  |  |  |  |
| **Control mortality** |  |  |  |
| <10% | 89.4% | 95.7% | 64.4% |
| ≥10% | ***10.6%*** | ***4.3%*** | ***35.6%*** |
|  |  |  |  |
| **Chemical effect class** |  |  |  |
| <20% | 36.3% | 47.7% | 30.2% |
| ≥20% and <40% | 32.7% | 29.4% | 29.2% |
| ≥40% | 31.0% | 22.8% | 40.9% |
|  |  |  |  |
| **Parasite effect class** |  |  |  |
| <20% | ***15.9%*** | ***14.2%*** | ***34.0%*** |
| ≥20% and <40% | 28.3% | 32.0% | 30.2% |
| ≥40% | 55.8% | 53.8% | 35.7% |
